# Chronic obstructive pulmonary disease candidate gene prioritization based on metabolic networks and functional information

**DOI:** 10.1371/journal.pone.0184299

**Published:** 2017-09-05

**Authors:** Xinyan Wang, Wan Li, Yihua Zhang, Yuyan Feng, Xilei Zhao, Yuehan He, Jun Zhang, Lina Chen

**Affiliations:** 1 Department of Respiratory, the Second Affiliated Hospital of Harbin Medical University, Harbin, Heilongjiang, China; 2 College of Bioinformatics Science and Technology, Harbin Medical University, Harbin, Heilongjiang, China; 3 Department of pharmacy, Heilongjiang Province Land Reclamation Headquarters General Hospital, Harbin, Heilongjiang, China; Imperial College London, UNITED KINGDOM

## Abstract

Chronic obstructive pulmonary disease (COPD) is a multi-factor disease, in which metabolic disturbances played important roles. In this paper, functional information was integrated into a COPD-related metabolic network to assess similarity between genes. Then a gene prioritization method was applied to the COPD-related metabolic network to prioritize COPD candidate genes. The gene prioritization method was superior to ToppGene and ToppNet in both literature validation and functional enrichment analysis. Top-ranked genes prioritized from the metabolic perspective with functional information could promote the better understanding about the molecular mechanism of this disease. Top 100 genes might be potential markers for diagnostic and effective therapies.

## Introduction

Chronic obstructive pulmonary disease (COPD) is the third leading cause of morbidity and mortality worldwide [1]. As a complex disease, COPD is caused by many factors, including smoking, advanced age, medications, systemic inflammation and especially metabolic disturbances [2]. For example, disturbances in glucose metabolism are more common in COPD patients than in COPD free individuals [3]. Schols found that COPD patients had an elevated energy metabolism [4]. Cathepsin S and cystatin C plasma levels were significantly higher in the COPD group than in the healthy group, and might serve as potential biomarkers for COPD [5].

Molecular changes occurring in the process of metabolism-related complex diseases could be represented in terms of metabolic networks [6], which have been used in many researches from various aspects. Shang et al. identified disease-related metabolites from a global metabolic network based on the assumption that the metabolites related to the same disease tend to be modularized in metabolic networks. Good performance and robustness were achieved for different disease classes, especially for respiratory diseases [7]. Oberhardt and colleagues integrated gene expression data with the metabolic network in Pseudomonas aeruginosa-infected chronic cystic fibrosis lung and demonstrated how the tradeoffs between growth and other important cellular processes shifted during disease progression [8]. Integrating other information into metabolic networks could help to better reveal disease mechanisms. Blais et al. manually curated metabolic networks to capture metabolic features. Then they integrated high-throughput transcriptomics data to predict biomarker changes in response to 76 environmental and pharmaceutical compounds for hepatocytes, which were validated with literature-based evidence and new experimental data [9]. Since genes with similar functions tend to be associated with similar diseases and vice versa [10–13], further investigation into COPD-related metabolic networks integrated with functional information is needed for better understanding of its mechanism.

Thus, in this paper, a gene prioritization method was applied to a COPD-related metabolic network, in which functional similarity was used to assess similarity between genes. Candidate genes in the COPD-related metabolic network were prioritized considering disease risks transferred between genes.

## Materials and methods

### Data

A human metabolic network was constructed by integrating interaction relationships from multiple databases, including the Human Metabolome Database (HMDB, http://www.hmdb.ca/) [14], HumanCYC (https://humancyc.org/) [15], BioGRID (https://thebiogrid.org/) [16], Reactome (http://www.reactome.org/) [17], Edinburgh Human Metabolic Network (EHMN) [18] and Kyoto Encyclopedia of Genes and Genomes (KEGG, http://www.genome.jp/kegg/) [19]. Protein IDs from these databases were converted to their corresponding gene official symbols. The integrated human metabolic network contained 5776 genes and 589199 interaction relationships between them.

29 COPD disease genes were obtained from Online Mendelian Inheritance in Man (OMIM, https://www.omim.org/) [20], the Disease Ontology (DO, http://disease-ontology.org/) [21], Phenotype-Genotype Integrator (PheGenI) (https://www.ncbi.nlm.nih.gov/gap/phegeni) [22], DISEASES (http://diseases.jensenlab.org/) [23] and Menche’s research [24].

Then, a COPD-related metabolic network was built using COPD disease genes and their direct interactors from the integrated human metabolic network. The COPD-related metabolic network was comprised of 6601 interactions (edges) between 1361 genes (nodes), 10 of which were COPD disease genes, and others were candidate genes.

Gene annotation information was collected from Gene Ontology (GO, http://www.geneontology.org/) [25]. All annotation terms for human genes in three ontologies, i.e. biological processes, molecular functions and cellular components, were extracted.

### Calculation of network weights

Network weights for the COPD-related metabolic network included two aspects: node (gene) weights and edge (interaction) weights.

The gene weight *w*_*g*_ for gene *g* was calculated as the fraction of GO terms annotated by *g* in all GO terms annotated by human genes:

$$w_{g}=\frac{\left|T_{g}\right|}{\left|T_{all}\right|}$$

where *T*_*g*_ represents GO terms annotated by *g* and *T*_*all*_ represents all GO terms annotated by human genes. |*X*| is the number of elements in the set *X*.

The interaction weight *w*_(*g*,*h*)_ was the functional similarity of two interacting genes *g* and *h*, as we defined in [26]:

$$w_{\left(g,h\right)}=\sum_{t\in T_{g}\cap T_{h}}\frac{1}{\left|G_{t}\right|}$$

where *T*_*g*_ and *T*_*h*_ are GO terms annotated by gene *g* and *h*, respectively. *G*_*t*_ is the set of genes annotated to a GO term *t*.

### Prioritization of candidate genes

The prioritization of candidate genes was performed based on disease risk scores of each gene obtained from an iteration process considering disease risks transferred between genes:

$$D^{\left(i+1\right)}=\left(1-\beta \right)QD^{\left(i\right)}+\beta D^{\left(0\right)}$$

where *D*^(*i*)^ is the vector of risk scores of all genes at step *i*, *β*∊(0,1) is a parameter to measure the importance between genes and interactions. After assessing the performance using *β* = 0.1, 0.2,⋯, 0.9, *β* = 0.1 was chosen as the optimal parameter.

*Q* is the disease risk transition probability matrix, which is composed of transition probabilities. The transition probability *q*(*g*|*h*) of disease risk going from gene *h* to gene *g* was defined as

$$q\left(g|h\right)=\frac{w_{\left(h,g\right)}}{\sum_{l\in neighbor\left(h\right)}w_{\left(l,g\right)}}$$

where *w*_(*h*,*g*)_ is the interaction weight between interacting genes *h* and *g*, *neighbor*(*h*) is the set of genes that interact with gene *h*.

*D*^(0)^ is the vector of initial disease risk scores for all genes, which was composed of scores *d*_*g*_^(0)^ for gene *g* in the COPD-related metabolic network:

$$d_{g}{}^{\left(0\right)}=\frac{w_{g}}{\sum_{m\in \text{COPD}-\text{related metabolic network}}w_{m}}$$

The iteration process was carried out until the difference between *D*^(*i*)^ and *D*^(*i*+1)^ was less than a threshold, 10^−9^. Candidate genes were prioritized based on their corresponding risk scores.

To further examine the functional relevance between the top-ranked genes and COPD, literature validation was performed for top 100 genes of the gene prioritization in literature of PubMed (http://www.ncbi.nlm.nih.gov/pubmed). Then, functional enrichment analysis was applied for top 100 genes using the Functional Annotation Tool in the Database for Annotation, Visualization and Integrated Discovery (DAVID, http://david.abcc.ncifcrf.gov/) v6.8 [27, 28]. GO functions and KEGG pathways with corrected P value (Benjamini) less than 0.05 were significant.

### Evaluation and comparison of the performance

Leave-one-out cross-validation (LOOCV) was carried out to assess the performance of the gene prioritization method. For all COPD disease genes, one gene was removed as a test gene at each time, and was added to candidate genes. The gene prioritization process was used to prioritize all the candidate genes. This process was repeated by setting every COPD disease gene to a test gene. The receiver operating characteristic (ROC) curves were plotted and the area under the curve (AUC) was computed based on the ranks of test genes. These results were compared with those of ToppGene and ToppNet using the same disease and candidate genes as our gene prioritization method did.

ToppGene and ToppNet are two tools in the ToppGene Suite (https://toppgene.cchmc.org) [29], which is an online bioinformatics tool for prioritizing candidate genes based on comprehensive factors, including GO annotation, phenotype, signaling pathway and protein interaction, from a set of genes known to be associated with the disease of interest.

Literature validation and functional enrichment analysis were also performed for top 100 genes of ToppGene and ToppNet to compare their efficiency with our gene prioritization method.

## Results

### Gene prioritization

COPD candidate genes were prioritized in the COPD-related metabolic network according to their risk scores in descending order. The top-ranked genes were more likely to be related to COPD. To further illustrate their correlation with COPD, literature validation and functional enrichment analysis were applied for top 100 genes (S1 Table).

In these genes, 56% (56/100) have been validated by literature. Higher proportion of validation was achieved for higher ranked genes. For example, 66% of top 50 genes and 90% of top 10 genes were validated to be associated with COPD by literature. For the first ranked gene CYP2E1, its polymorphisms were found to be over-represented in COPD patients [30]. Protein levels of SOD1 (rank: 3) were significantly higher in both tumor and non-tumor lung specimens of COPD patients than in lung cancer patients with no COPD. This result indicated that SOD1 could participate in antioxidant defense of the lungs in COPD patients [31]. Genetic variations in enzyme-coding genes CYP2C9 (rank: 4) and CYP1B1 (rank: 8) have shown potentially risk of tobacco-related diseases, including COPD. Their corresponding enzymes metabolize polycyclic aromatic hydrocarbons found in tobacco smoke and generate disease-causing metabolites [32]. The SNP rs2682825 in the gene NOS1 (rank: 5) was revealed to be associated with qualitative COPD phenotypes [33].

**Fig 1:**
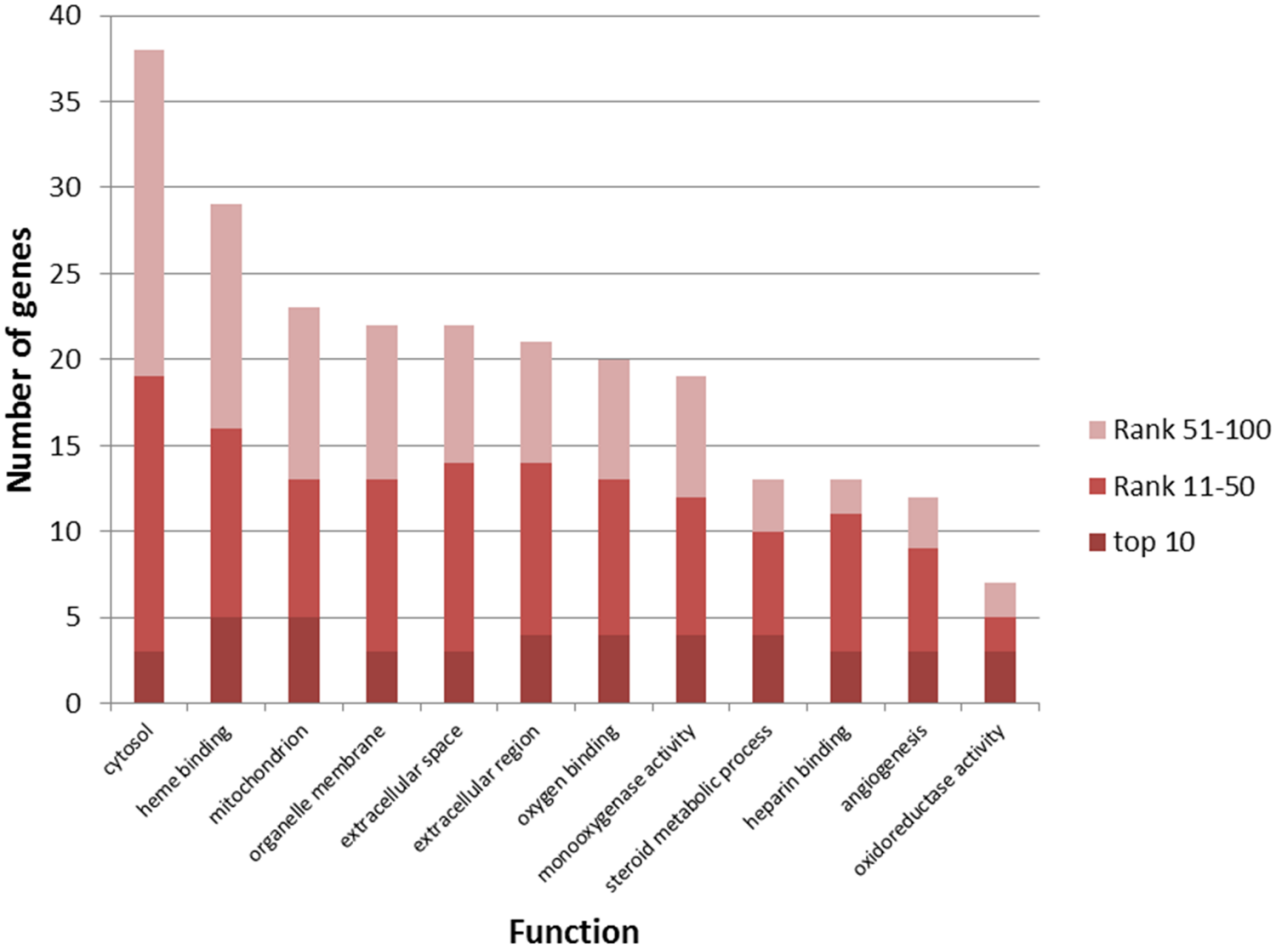
Some of COPD-related GO functions significantly enriched by top 100 genes. GO functions (horizontal axis) were significantly enriched by top 100 genes (the number in the vertical axis) using DAVID (Benjamini corrected P value<0.05).

Top 100 genes were significantly enriched in 143 GO functions, 86 (60.140%) of which were annotated by COPD disease genes and regarded as COPD-related functions (Some are illustrated in Fig 1). “Heme binding” is a process through which the enzyme heme oxygenase-1 catalyzed the oxidative degradation of heme to play a protective role as an antioxidant in the lung [34]. The promoter polymorphism of the gene coding for the enzyme has been shown to be associated with the severity and prognosis of COPD patients [35]. Fathy et al. found that “angiogenesis” was significantly decreased among COPD patients compared with controls after evaluating angiogenesis by counting microvessels highlighted using anti-CD34 antibody as a measure of microvascular density [36]. Busch et al. found five differentially methylated CpG probes significantly associated with COPD among African-Americans. The top differentially methylated CpG site was mapped to the gene MAML1, which affected NOTCH-dependent “angiogenesis” in lungs [37]. Recent researches indicated that Gram-negative bacteria-derived vesicles in “extracellular regions” could evoke neutrophilic pulmonary inflammation, a key pathology of COPD [38]. Levels of several damage-associated molecular patterns were also increased in lung fluids, the lung “extracellular region”, of COPD patients [39].

**Fig 2:**
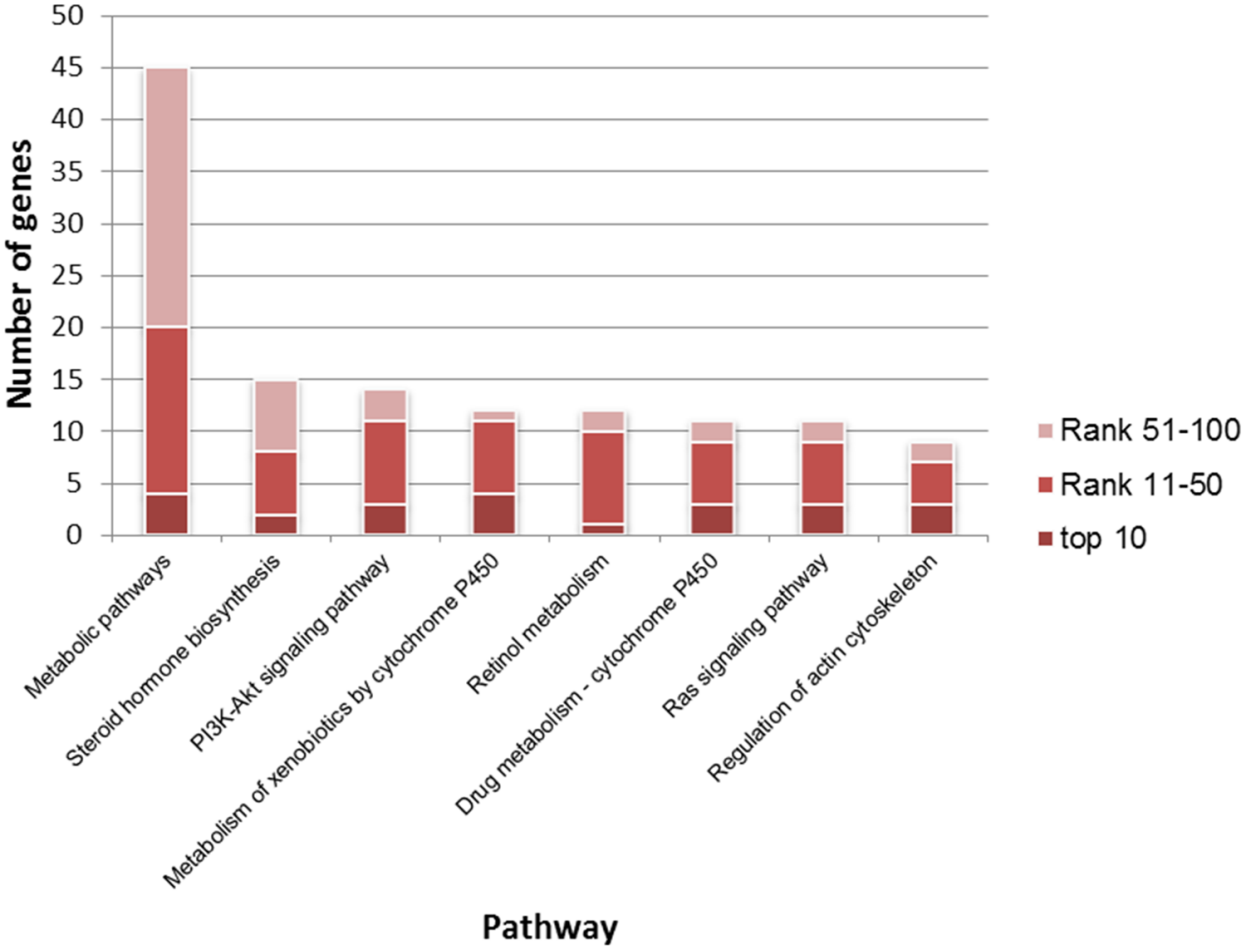
Some of COPD-related KEGG pathways significantly enriched by top 100 genes. KEGG pathways (horizontal axis) were significantly enriched by top 100 genes (the number in the vertical axis) using DAVID (Benjamini corrected P value<0.05).

**Fig 3:**
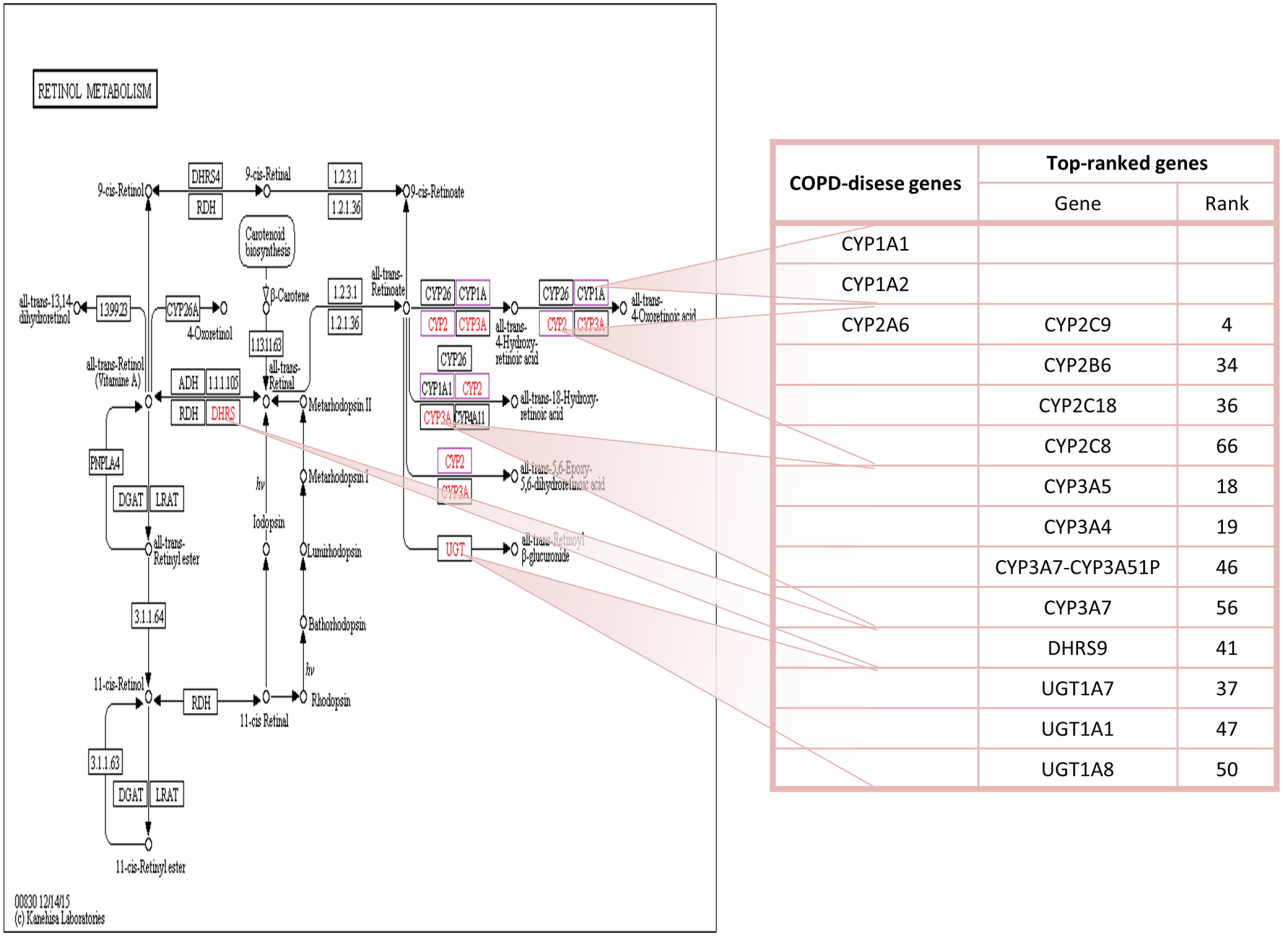
The retinol metabolism pathway and its top-ranked genes. Nodes with red name contain top 100 genes, whose ranks are listed in the right table. Nodes with purple border contain COPD-disease genes.

34 KEGG pathways were significantly enriched by top 100 genes. 32 (94.118%) were COPD-related pathways that were annotated by COPD disease genes (Some are illustrated in Fig 2). “Metabolic pathways” was enriched by the most genes. COPD has been linked to the dysregulation of many “metabolic pathways”, such as “Steroid hormone biosynthesis”. These metabolic pathways might be useful targets for novel COPD therapies [40, 41]. “Steroid hormone biosynthesis” was associated with COPD since steroid hormones are involved in lung development, pulmonary inflammation, and lung cancer. Signaling and exposure of estrogen, a group of steroid hormones, played a role in pulmonary disorders, including COPD [42]. Inflammation caused by COPD could be reduced by enhancing the anti-inflammatory effects of steroids [43]. “Metabolism of xenobiotics by Cytochrome P450” was significantly regulated by a set of genes in regulating inflammatory airway diseases, such as COPD [44]. HoffMann et al. found that “retinol metabolism” (Fig 3) was the most significantly differentially regulated pathway between pulmonary hypertension patients with COPD and idiopathic pulmonary fibrosis. They also pointed out that genes related to “retinol metabolism” might play an important role in differentiating processes involved in vascular remodeling of pulmonary hypertension caused by COPD and other lung diseases [45]. Signaling pathways were also involved in COPD. The “PI3K/Akt signaling pathway” is required for urokinase plasminogen activator receptor-mediated Epithelial-mesenchymal transition in human small airway epithelial cells, which played a crucial role in small airway fibrosis of COPD patients [46].

**Fig 4:**
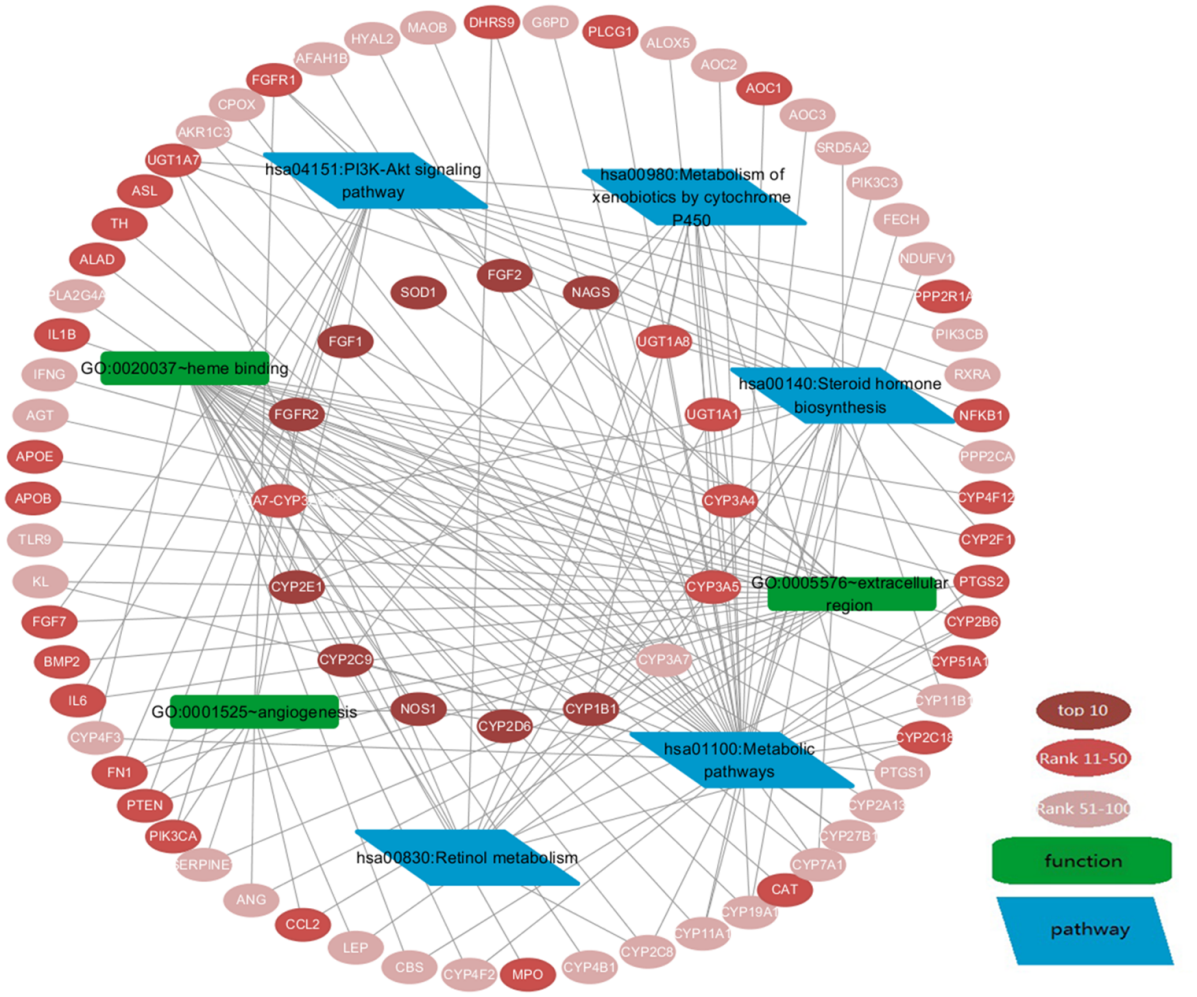
Top 100 genes and some of their enriched COPD-related functions or pathways. GO functions (green round rectangle) and KEGG pathways (blue parallelogram) were significantly enriched by top 100 genes (red ellipse) using DAVID (Benjamini corrected P value<0.05).

Functional enrichment analysis demonstrated the correlation of COPD and top 100 genes, most of which have been validated by literature. Other genes without literature validation could also be enriched in these COPD-related functions or pathways. For example, CYP51A1 (rank: 12), UGT1A7 (rank: 37) and FN1 (rank: 38) were annotated to “heme binding” function, “Metabolism of xenobiotics by cytochrome P450” pathways and “PI3K-Akt signaling pathway”, respectively (Fig 4).

### Performance evaluation and comparison

**Fig 5:**
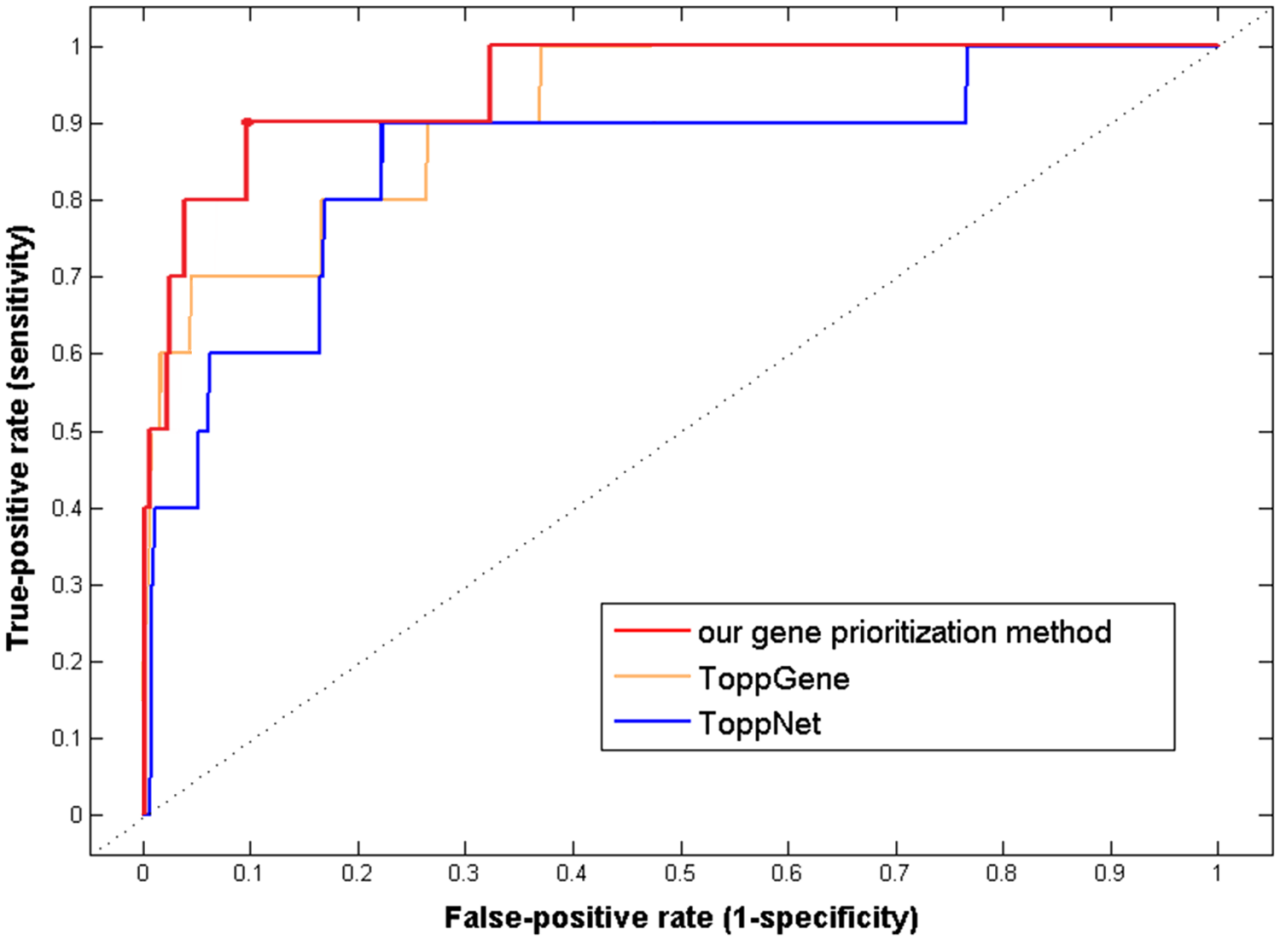
The ROC curves of our gene prioritization method, ToppGene and ToppNet.

The gene prioritization performance was assessed using LOOCV, AUC of which was compared with that of ToppGene and ToppNet (Fig 5). It was showed that AUC of our gene prioritization method was 0.949, which was higher than that of both ToppGene (0.912) and ToppNet (0.854).

**Fig 6:**
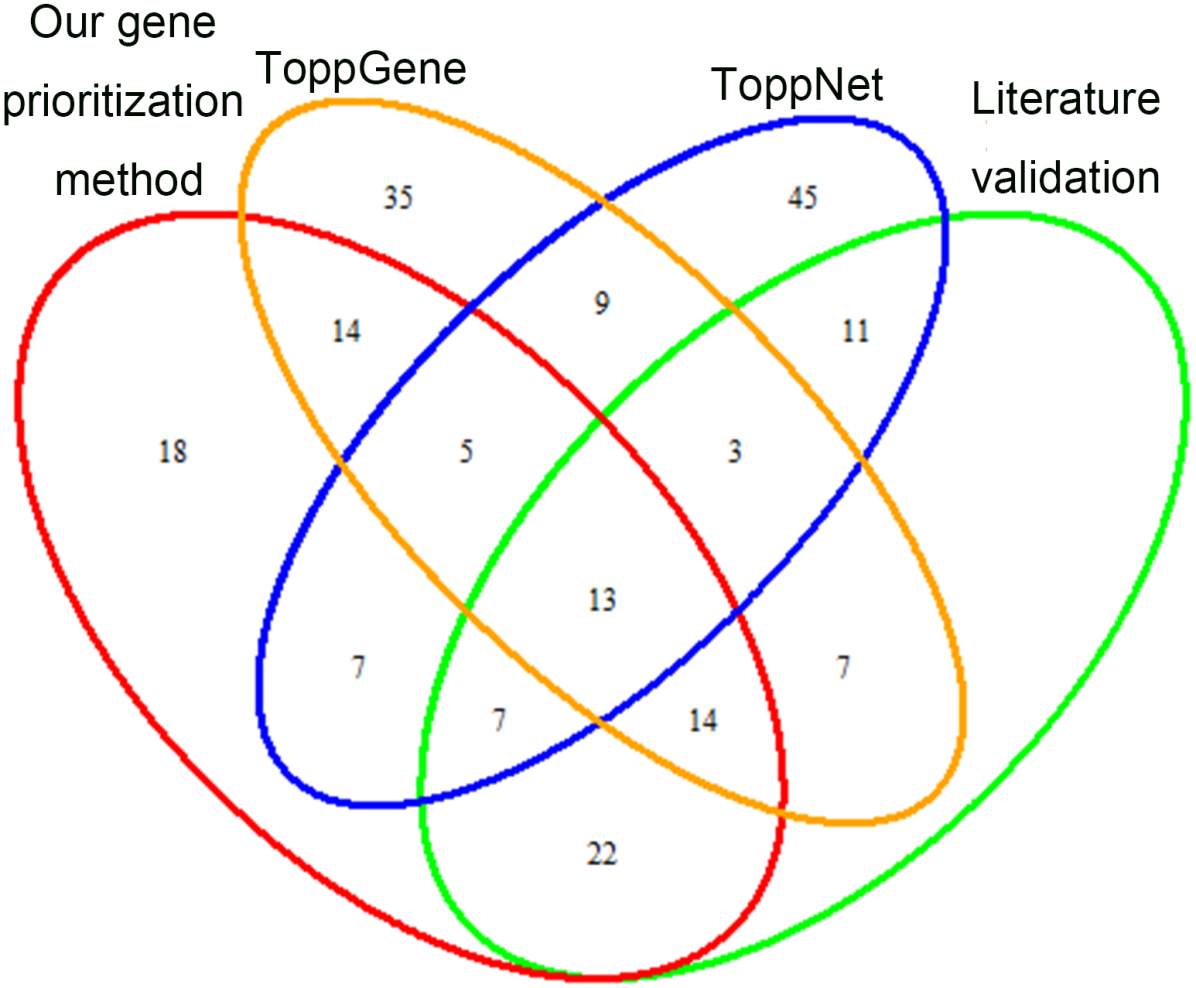
Overlap of top 100 genes from our gene prioritization method, ToppGene and ToppNet, and their literature validation.

Then the performance of the three methods was compared on their literature validation (Fig 6). For ToppGene, 37% of its top 100, 42% of its top 50, and 70% of its top 10 genes were validated, while for ToppNet, 34% of top 100, 36% of top 50, and 50% of top 10 were validated to be involved in COPD. All of these proportions were less than those of our gene prioritization method (56%, 66% and 90%).

The performance of the three methods was also compared on COPD-related function or pathway proportion of top 100 genes (Table 1). Results of functional enrichment analysis showed that 100 and 75 functions, as well as 20 and 19 pathways were significantly enriched by top 100 genes of ToppGene and ToppNet, respectively. In these functions and pathways, 55% (55/100) and 56% (42/75) of functions, and 75% (15/20) and 47.368% (9/19) pathways were COPD-related. The numbers and proportions were both less than those of our gene prioritization method. That is, functions and pathways significantly enriched by top genes of our gene prioritization method were more associated with COPD, which indicated that our top genes were more likely to be related to COPD.

**Table 1:** The number of significantly enriched functions and pathways, and the number and proportion of COPD-related functions and pathways in these significantly enriched functions and pathways, for top 100 genes from our gene prioritization method, ToppGene and ToppNet.

	Function		Pathway	
	Significantly enriched	COPD-related (proportion)	Significantly enriched	COPD-related (proportion)
**Our gene prioritization method**	143	86 (60.140%)	34	32 (94.118%)
**ToppGene**	100	55 (55%)	20	15 (75%)
**ToppNet**	75	42 (56%)	19	9 (47.368%)

These results demonstrated the good performance of our gene prioritization method, which was superior to both ToppGene and ToppNet.

## Discussion

In this paper, a gene prioritization method was applied to a COPD-related metabolic network to prioritize COPD candidate genes according to their risk scores in descending order. Literature validation and functional enrichment analysis were assessed for top 100 genes. The performance of the gene prioritization method was better on AUC of LOOCV, literature validation and COPD-related function or pathway proportion of top 100 genes than those of ToppGene and ToppNet.

To further exhibit the performance of our gene prioritization method, a linear support vector machine classifier was applied to classify samples of an expression profile GSE57148 from Gene Expression Omnibus (GEO, https://www.ncbi.nlm.nih.gov/geo/) [47] for top 10 (the same number as COPD disease genes in the COPD-related metabolic network), top 29 (the same number as all COPD disease genes), and top 100 genes, respectively. The profile contained 98 COPD patients and 91 normal controls. To assess the performance of our top-ranked genes, the same classification process was also conducted for 10 COPD disease genes in the COPD-related metabolic network and 29 COPD disease genes from multiple databases (see Data). AUC was used to compare their classification performance. The classification performance of top 10 genes of our gene prioritization method (0.729) was slightly better than that of 10 COPD disease genes (0.725), while the classification performance of 29 COPD disease genes (0.837) was better than that of top 29 genes of our gene prioritization method (0.810). Top 100 genes could classify samples with high AUC (0.789). It was shown that, like COPD disease genes, top 100 genes could classify samples with good performance.

Top-ranked genes prioritized from the COPD-related metabolic network could be significantly enriched in some metabolic pathways, including “Metabolism of xenobiotics by cytochrome P450” and “Drug metabolism—cytochrome P450”. In these pathways, cytochrome P450, essential enzymes for the metabolism of many medications, was involved. Besides, 23 genes in top 100 genes and 3 COPD disease genes (CYP1A1, CYP1A2 and CYP2A6) participate in components of cytochrome P450. Goblet cell-associated cytochrome P450 activity elevated leukotoxin-diol levels, which played a role in the clinical manifestations of COPD in a female-dominated disease sub-phenotype [48]. Plasma epoxyeicosatrienoic acids synthesized by cytochrome P450 enzymes and produced in lung epithelial cells might become dysfunctional in COPD because of the synergistic effect caused by smoking with cytochrome P450 polymorphisms [49].

Top-ranked genes should also be validated in GWAS data, since some of our disease genes were from the PheGenI, which merges NHGRI GWAS catalog data. We first searched public databases storing genes corresponding to GWAS results, including ClinVar [50], GWAS Central [51] and GWASdb [52]. Three genes were in top 200 genes of our prioritization method: FGF7 (Rank: 34), ACE (Rank: 115) and SLC6A4 (Rank: 123), all of which were validated in literature. Additionally, GWAS were performed for genotype data we retrieved from GSE57148, a high throughput sequencing dataset from lung tissues of COPD patients versus normal controls in GEO. Disease significantly associated SNPs and SNPs in high linkage disequilibrium with them were considered (P-value<5x10^-8^). Another three genes mapped to these SNPs were in top 200 genes of our prioritization method, i.e. PTPRJ (Rank: 104), PLCG2 (Rank: 109) and ATP2B4 (Rank: 137).

The gene prioritization method we proposed here could prioritize COPD candidate genes with a good performance. However, only 6 genes of our top 200 genes could be obtained by GWAS. This implied that our top-ranked genes could be a complement to GWAS data, and our method depended on disease genes and the disease-related network based on these genes. With the intrinsic limitation of the COPD-related metabolic network, some genes involved in COPD might be filtered out. To make up this efficiency, novel information of disease genes and the metabolic network should be added and considered comprehensively.

## Conclusions

COPD candidate genes were prioritized in a COPD-related metabolic network using the gene prioritization method. The correlation of COPD and top 100 genes was validated by literature and functional enrichment analysis. The performance of the gene prioritization method was better than ToppGene and ToppNet. In summary, top-ranked genes prioritized from the metabolic perspective with functional information could promote the better understanding about the molecular mechanism of this disease. Top 100 genes might act as potential markers for diagnostic and effective therapies.

## Supporting information

S1 Table Top 100 genes of our gene prioritization method and PMIDs for their correlations with COPD. (DOC)
