# Supplementary material for: Chronic obstructive pulmonary disease candidate gene prioritization based on metabolic networks and functional information
Source: PLoS One. 2017 Sep 5;12(9):e0184299. doi: 10.1371/journal.pone.0184299 (PMC5584748; doi:10.1371/journal.pone.0184299)
Supplement: S1 Table — (DOC) [file pone.0184299.s001.doc]

**S1 Table. Top 100 genes of our gene prioritization method and PMIDs for their correlations with COPD.**

| **Rank** | **Top 100 genes** | **PMIDs** |
| --- | --- | --- |
| 1 | CYP2E1 | 17442289 |
| 2 | CYP2D6 | 23881566 |
| 3 | SOD1 | 19705749 |
| 4 | CYP2C9 | 22272893 |
| 5 | NOS1 | 25043200, 24192154 |
| 6 | FGFR2 | 24730525 |
| 7 | FGF2 | 22700853 |
| 8 | CYP1B1 | 22272893, 20198857 |
| 9 | NAGS | - |
| 10 | FGF1 | 20970515 |
| 11 | PIK3CA | 26617776 |
| 12 | CYP51A1 | - |
| 13 | FGFR1 | 15772985 |
| 14 | MPO | 24588870 |
| 15 | TH | - |
| 16 | PTPN11 | - |
| 17 | ESR1 | 27940297, 21625484 |
| 18 | CYP3A5 | 24535486 |
| 19 | CYP3A4 | 28161533 |
| 20 | PTEN | 19625176 |
| 21 | PLCG1 | - |
| 22 | ALAD | - |
| 23 | CCL2 | 24697203 |
| 24 | NFKB1 | 27003425, 23322360 |
| 25 | IL1B | 26617776 |
| 26 | CYP2F1 | 23849338 |
| 27 | IL6 | 17380888 |
| 28 | ASL | 25795727 |
| 29 | CAT | 25018154 |
| 30 | APOE | 24096154 |
| 31 | AR | - |
| 32 | CYP2B6 | 19251795 |
| 33 | HDAC4 | 27793800 |
| 34 | FGF7 | 22796760 |
| 35 | SOD2 | 16467073 |
| 36 | CYP2C18 | - |
| 37 | UGT1A7 | - |
| 38 | FN1 | - |
| 39 | PPP2R1A | - |
| 40 | BMP2 | - |
| 41 | DHRS9 | - |
| 42 | APOB | 26137575 |
| 43 | AOC1 | - |
| 44 | RARA | 24111541 |
| 45 | CYP4F12 | - |
| 46 | CYP3A7-CYP3A51P | - |
| 47 | UGT1A1 | 24295085 |
| 48 | PTGS2 | 25767384 |
| 49 | PTPN1 | 24587397 |
| 50 | UGT1A8 | - |
| 51 | PPP1CA | - |
| 52 | G6PD | 23277075 |
| 53 | AOC3 | 27932762 |
| 54 | MAOB | - |
| 55 | CYP2A13 | - |
| 56 | CYP3A7 | - |
| 57 | AGT | 26064378 |
| 58 | PPP2CA | - |
| 59 | LYPLA1 | - |
| 60 | GJA1 | - |
| 61 | DNM2 | - |
| 62 | PTPN6 | - |
| 63 | CYP27B1 | 28161533 |
| 64 | PPARG | 25634111 |
| 65 | PAFAH1B1 | - |
| 66 | CYP2C8 | - |
| 67 | NDUFV1 | - |
| 68 | RXRA | 28161533 |
| 69 | SERPINE1 | 27856929 |
| 70 | LEP | 20854423 |
| 71 | CYP11A1 | - |
| 72 | FECH | - |
| 73 | ALOX5 | - |
| 74 | SRD5A2 | - |
| 75 | ADRB2 | 22383665 |
| 76 | PIK3CB | - |
| 77 | CBS | 26455818 |
| 78 | KL | 26201096 |
| 79 | CYP19A1 | 27940297 |
| 80 | CPOX | 25424692 |
| 81 | AKR1C3 | 23665002 |
| 82 | PIK3C3 | - |
| 83 | ATP1A1 | - |
| 84 | SULT1C2 | - |
| 85 | AOC2 | - |
| 86 | USP7 | 21455491 |
| 87 | IFNG | 26403459 |
| 88 | PLA2G4A | 25634111 |
| 89 | HYAL2 | 20864512 |
| 90 | PTGS1 | - |
| 91 | ATP1B1 | - |
| 92 | CYP4F3 | - |
| 93 | CYP4B1 | 19352772 |
| 94 | CYP7A1 | - |
| 95 | ICAM1 | 22349390 |
| 96 | CYP4F2 | - |
| 97 | CYP11B1 | - |
| 98 | TLR9 | 22836641 |
| 99 | DNM1L | 25083992 |
| 100 | ANG | 26755908 |
